# Cunningham's skinks show low genetic connectivity and signatures of divergent selection across its distribution

**DOI:** 10.1002/ece3.2627

**Published:** 2016-11-29

**Authors:** Benjamin Y. Ofori, Linda J. Beaumont, Adam J. Stow

**Affiliations:** ^1^ Department of Biological SciencesMacquarie UniversityNorth RydeMacquarie ParkNSWAustralia; ^2^ Department Animal Biology and Conservation ScienceUniversity of GhanaLegon‐AccraGhana

**Keywords:** adaptive genetic variation, conservation genetics, *Egernia cunninghami*, local adaptation, next‐generation sequencing, single nucleotide polymorphisms

## Abstract

Establishing corridors of connecting habitat has become a mainstay conservation strategy to maintain gene flow and facilitate climate‐driven range shifts. Yet, little attention has been given to ascertaining the extent to which corridors will benefit philopatric species, which might exhibit localized adaptation. Measures of genetic connectivity and adaptive genetic variation across species’ ranges can help fill this knowledge gap. Here, we characterized the spatial genetic structure of Cunningham's skink (*Egernia cunninghami*), a philopatric species distributed along Australia's Great Dividing Range, and assessed evidence of localized adaptation. Analysis of 4,274 SNPs from 94 individuals sampled at four localities spanning 500 km and 4° of latitude revealed strong genetic structuring at neutral loci (mean *F*_ST_ ± *SD* = 0.603 ± 0.237) among the localities. Putatively neutral SNPs and those under divergent selection yielded contrasting spatial patterns, with the latter identifying two genetically distinct clusters. Given low genetic connectivity of the four localities, we suggest that the natural movement rate of this species is insufficient to keep pace with spatial shifts to its climate envelope, irrespective of habitat availability. In addition, our finding of localized adaptation highlights the risk of outbreeding depression should the translocation of individuals be adopted as a conservation management strategy.

## 1 Introduction

The threat of climate change to global biodiversity is a major focus of conservation‐based research and management (Loss, Terwilliger, & Peterson, 2011). Mountain ecosystems have received particular attention because they harbor higher proportions of endemic species, and these species face increased risk of extinction because of their narrow thermal tolerance and elevational ranges (Bell, Bradford, & Lauenroth, 2010; Elsen & Tingley, 2015; Frei et al., 2014). The persistence of montane species as climate changes depends on their ability to shift their ranges to higher latitudes and altitudes (Chen et al., 2011), or adapt to future climatic conditions (Hoffmann & Sgrò, 2011). However, altitudinal range shifts may be constrained by limited upslope area and movement restrictions imposed by topography and habitat fragmentation (Bell et al., 2010; Elsen & Tingley, 2015). Further, because montane species typically have small, multiple disjunct populations (Huntley & Barnard, 2012; Sgro, Lowe, & Hoffmann, 2011), they may lack the capacity to adapt rapidly enough to counter the speed and magnitude of contemporary climate change. Therefore, these species may require management to increase their resilience and adaptive capacity.

Creating and maintaining habitat corridors is one of the most appealing and politically favoured strategies for conserving montane species (Pulsford et al., 2013). The rationale behind this strategy is that increasing habitat connectivity along mountain ranges facilitates range shifts by enabling individuals to track the movement of their climatic envelope, thereby enhancing exchange of individuals and genes among metapopulations, increasing effective population sizes and adaptive potential (Steffen et al., 2009). Corridors could help mediate the ecological and evolutionary processes necessary to sustain communities under changing environments (Doerr, Barrett, & Doerr, 2011).

While the importance of corridors has been demonstrated for wide‐ranging and highly mobile species (Heller & Zavaleta, 2009; Sharma et al., 2013), their capacity to facilitate range shifts and enhance the adaptive potential of philopatric species is debatable (Beier & Noss, 1998; Hodgson et al., 2009). Species with low vagility may be unable to undertake the rapid long‐distance dispersal necessary to accommodate climate change (Broquet & Petit, 2009). However, it is generally agreed that corridors can provide stepping stones of high‐quality breeding habitat for philopatric species. This should allow them to undertake multigenerational range shifts, with potential for localized gene flow (Hodgson et al., 2009). The extent to which this is the case in montane ecosystems, where suitable habitat is often highly fragmented, remains unclear.

Limited gene flow between localities, in addition to the effects of drift and selection, will lead to strong genetic differentiation and, potentially, local adaptation (Nosil, Funk, & Ortiz‐Barrientos, 2009). Facilitating gene flow between species with high genetic structuring and divergence may be problematic as this can lead to disruption of locally adapted gene complexes and result in outbreeding depression (Frankham et al., 2011; Sexton, Strauss, & Rice, 2011; Slatkin, 1987). Other nonadaptive genetic processes, such as chromosomal rearrangements, can also contribute to outbreeding depression (Frankham et al., 2011). Thus, it is important to identify reproductively isolated and locally adapted populations in order to delineate conservation units for effective management (Moore et al., 2014). In this regard, measures of genetic variation at both neutral and loci associated with adaptation are required.

For most conservation‐oriented studies, patterns of genetic structure and local adaptations have been characterized using neutral markers. Markers such as microsatellite genotypes can reveal the patterns of gene flow and reproductively isolated populations. However, because they are selectively neutral (Lowe & Allendorf, 2010; Nosil et al., 2009), they do not reveal geographical patterns of adaptive genetic variation or the scale of local adaption (Allendorf, Hohenlohe, & Luikart, 2010; Sheth & Angert, 2016). Although neutral and adaptive genetic variation may sometimes show similar spatial patterns (Moore et al., 2014), divergence at neutral and adaptive loci arises principally from different processes. Divergence at neutral loci arises from limited dispersal and gene flow, and genetic drift, whereas adaptive divergence arises from selective sweeps which rapidly increase the frequency of a favoured allele due to directional selection pressures (Frankham, Briscoe, & Ballou, 2002; Lowe & Allendorf, 2010; Nosil et al., 2009; Sexton et al., 2011; Slatkin, 1987).

In a homogeneous environment, species with limited dispersal capacity and small population sizes may show considerable genetic partitioning at neutral loci, but may not be differentiated at adaptive loci (Sexton, Hangartner, & Hoffmann, 2014). In a heterogeneous environment, spatial patterns of neutral and adaptive genetic variation may vary depending on the limits of dispersal, and the strength and spatial gradient of selection pressures (Forester et al., 2016; Manel & Holderegger, 2013). As a result, spatial patterns of neutral genetic variation may not be an adequate proxy for adaptive genetic variation and local adaptation, necessitating the need to investigate the spatial patterns of both types of genetic variations.

Recent advances in next‐generation sequencing technologies have increased the availability of genomic data in nonmodel species, making it possible to identify loci that are under divergent selection or are physically linked to regions of the genome that are under divergent selection (Allendorf et al., 2010; Hess et al., 2013; Nosil et al., 2009). Genome scanning has highlighted the spatial patterns of adaptive genetic variation and local adaptations associated with selection in nonmodel species (Forester et al., 2016; Schweizer et al., 2015). Such knowledge can highlight the potential benefits and risks of enhancing gene flow between populations from dissimilar environments and inform conservation actions under rapid climate change.

The Great Dividing Range (GDR) of Australia is a key conservation area, and harbors globally endemic and endangered species (Steffen et al., 2009). This mountain range traverses almost 3,500 km of the Australian continent, running from the Alps in southeast Victorian to Atherton in northeast Queensland (Pepper et al., 2014). In 2007, the Australian Government, through the Environment Heritage and Protection Council (EPHC), announced plans to create a connectivity corridor along the GDR to enhance species’ range shifts and resilience to climate change (Worboys & Pulsford, 2011).

Similar large‐scale conservation corridors have also been established elsewhere, such as the Yellowstone to Yukon project (Y2Y) in North America. This connects the northern Rocky Mountains of the USA and Canada (Graumlich & Francis, 2010). Other examples include the Albertine Rift connectivity in Africa that runs through DR Congo, Rwanda, and Uganda (Plumptre et al., 2007), the Condor Biosphere reserve connectivity in the Ecuadorian Andes (Benitez & Cuesta, 2003), and the Catalonia area of northeast Spain and connectivity to the European Alps (Rafa, 2004). However, the Australian GDR remains the first continental‐scale corridor (Worboys & Pulsford, 2011).

Here, we use Cunningham's skink (*Egernia cunninghami*) as a model philopatric species to describe spatial patterns of neutral and adaptive genetic variation along the GDR. Reptiles in general have received less attention in the climate change literature, despite being relatively more sensitive to climate change than other vertebrate taxa, such as birds and mammals (Cabrelli, Stow, & Hughes, 2014; Sinervo et al., 2010). In Australia and within the GDR, reptiles are the most diverse and dominant vertebrates, and about 7% of them are listed as threatened under State Acts (Chapman, 2009; Steffen et al., 2009). *Egernia cunninghami* is a common scincid lizard distributed along the GDR and coastal strips to the east. The species is protected throughout its distribution and is declared as threatened in Southern Australia, where some isolated populations have gone locally extinct. Typical of philopatric species, it shows localized movement and high retreat‐site fidelity (Stow & Sunnucks, 2004), and its ecology and basic biology are well researched (Bickford et al., 2010; Brown, 1991; Chapple, 2003; Kearney et al., 2013; Stow et al., 2001).

Our main goals are to assess the levels of gene flow and evidence for selection among four populations of Cunningham's skink that are separated by ~100–500 km. Specifically, we ask: (1) Is localized philopatry in *E. cunninghami* reflected by strong genetic partitioning among regions? (2) Is genetic structure at neutral loci a good proxy for adaptive variation? We discuss the implications of our results for mitigating negative impacts of climate change on this species.

## 2 Materials and Methods

### 2.1 Tissue sample collection and DNA extraction

**Figure 1:**
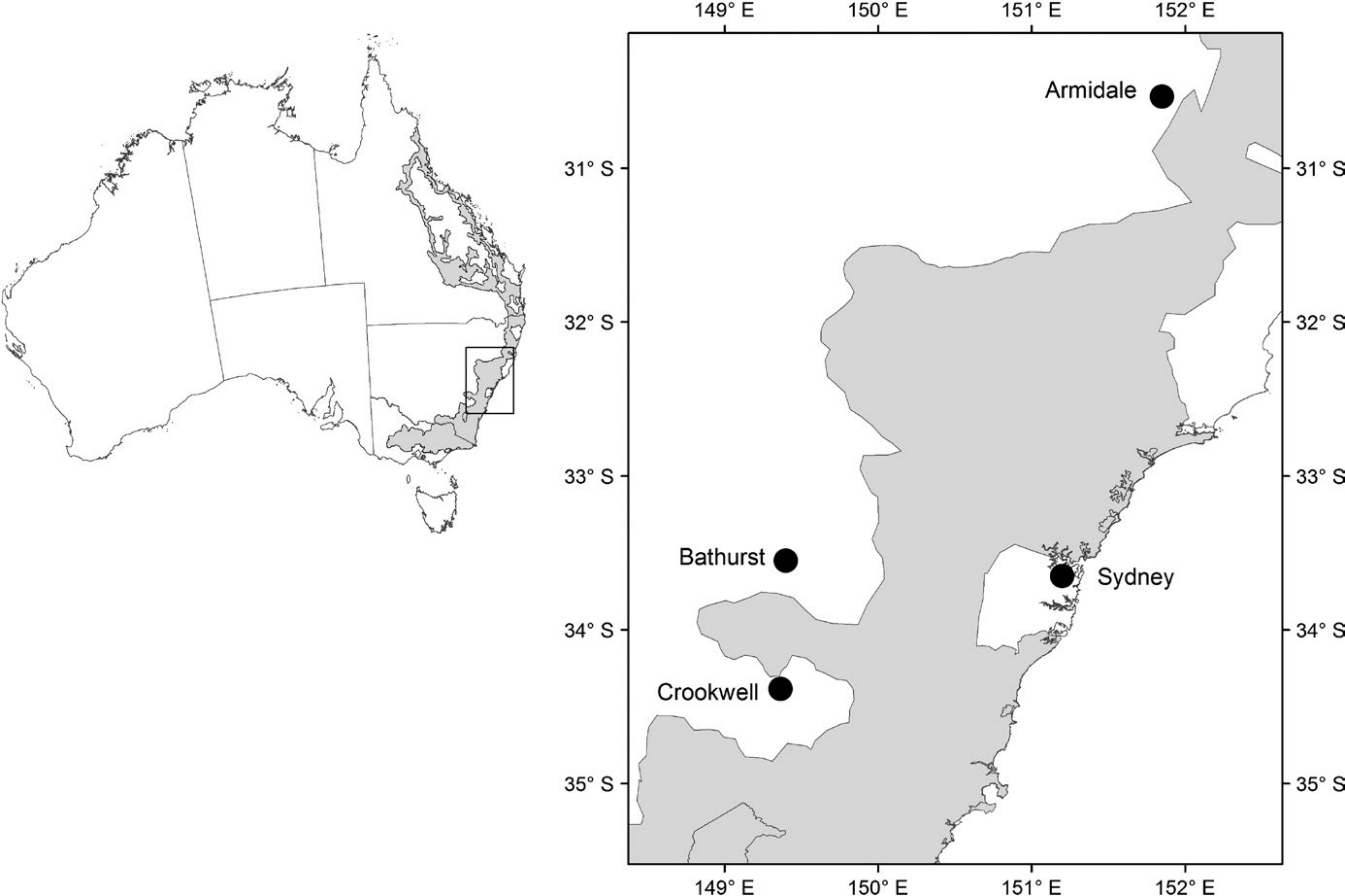
Sampling localities of Cunningham's skink (*Egernia cunninghami*) along the Great Dividing Range, southeastern Australia. The Great Eastern Range Connectivity Corridor is highlighted in gray

Tail‐tip muscle tissue samples of Cunningham's skink were collected from 94 individuals at four localities across the GDR within New South Wales, Australia (Figure 1). We sampled 18 individuals at Sydney (33°39′ S, 151° 12′), 27 at Armidale (30° 32′ S, 151° 51′ E) in the Northern Tablelands, 27 at Bathurst (33° 33′ S, 149° 24′ E) and 22 at Crookwell (34° 23′S, 149° 22′) in the Central Tablelands. The Euclidean distance between pairs of localities ranged from 96 km (Bathurst vs. Crookwell) to 500 km (Armidale vs. Crookwell; Table S1).

Individuals were captured using Elliott live traps and by hand. Captured individuals were measured and released at the point of capture after a small terminal portion of the tail was removed. The exact locations where samples were taken were marked using a global positioning system (GPS) unit (Garmin GPSmap 62). Tissue samples were kept in 97% ethanol at room temperature prior to laboratory analysis. Lizard capturing and handling followed Macquarie University Animal Ethics Committee recommendations (ARA 2013/015) and was licensed by the Office of Environment & Heritage, NSW National Parks and Wildlife Service (SL101164).

### 2.2 DNA extraction, SNP discovery, and screening

Genomic DNA was extracted from tissue using a commercially available spin‐column kit (GenCatch^™^ Blood & Tissue Genomic DNA Extraction Mini‐Prep Kit; Epoch Life Science, Inc.) following manufacturer's protocols. SNP discovery and genotyping were performed at Diversity Arrays Technology Pty. Ltd. (Canberra, Australia) using standard DartSeq^™^ protocol (for details of the SNPs discovery and screening protocol, see Text File S1). Discovered SNPs were called only if they were present in both homozygous and heterozygous forms.

To ensure the quality of individual samples, all duplicate markers and those with minor allele frequencies <5% (MAF < 0.05) across all populations were removed. We also removed markers with average read depth <10 or >45 for both alleles; individuals genotyped at <100% call rate (CR: proportion of genotyped SNPs); <94% reproducibility and two or more SNPs that occurred on one sequence read to avoid physical linkage. DArT sequencing returned a total of 81,732 SNPs, of which 4,274 were retained for downstream analyses after secondary filtering. DNA sequences and statistics (i.e., call rate, polymorphic information content, heterozygosity, read depth, and reproducibility for all loci and individuals) are accessible from the Dryad Digital Repository (http://dx.doi.org/10.5061/dryad.ko1kj) and Diversity Array Technology Pty. Ltd., Canberra, Australia (Report‐DEgs14‐1547).

### 2.3 Detection of SNPs under divergent selection (putatively adaptive SNPs)

We used three alternative methods to identify loci putatively under divergent selection from the 4,274 unique SNPs retained for analysis. These approaches are based on outlier loci, defining loci under divergent selection as those with greater than the expected levels of divergence among regional groups, and loci under balancing selection as those with smaller than expected levels of divergence (*F*_ST_) among regional groups (Moore et al., 2014). First, we used BAYESCAN, a Bayesian approach that estimates the posterior probability of a given locus being under selection (Foll & Gaggiotti, 2008). We ran BAYESCAN using the default settings as test runs with longer chain parameters gave identical results. Loci under divergent selection were defined as those with greater *F*_ST_ than the mean among the group and α‐values significantly >0. Loci with *F*_ST_ smaller than expected among the group and α‐values significantly less than 0 were considered as balancing selection. All other loci were considered putatively neutral.

Second, we used the hierarchical island model implemented in Arlequin v.3.5 (Excoffier & Lischer, 2010). This method allows lower migration rates among groups to be compared to within groups, and has been shown to reduce the prevalence of false positive (Excoffier & Lischer, 2010). We ran 20,000 simulations with 100 demes per group for 10 groups. Loci with *F*_ST_ significantly (*p *≤* *.01) higher than the mean were considered candidates for divergent selection and those with *F*_ST_ significantly less than the mean among groups were considered candidates for balancing selection. Finally, we ran LOSITAN (Antao et al., 2008) using parameter settings of 50,000 simulations, confidence interval of 0.99, FDR of 0.1, and subsample size of 49. Again, we identified loci under divergent selection as those with significantly greater *F*_ST_ than the among group mean *F*_ST_ and balancing selection as loci with *F*_ST_ significantly smaller than this mean.

To examine gene ontology annotation terms associated with the SNPs under divergent selection, we BLASTed the trimmed sequences for all SNPs identified as candidates for divergent selection against the UNI‐PROT/SWISS‐PROT and NCBI nonredundant nucleotide database (Altschul et al., 1997). We set the BLAST *e*‐value acceptance threshold as 1 × 10^−6^ with a sequence homology of more than 70% (Benestan et al., 2016).

### 2.4 Summary statistics

We tested for deviation from Hardy–Weinberg equilibrium (HWE) at each sampling locality, and computed levels of expected (*H*_e_) and observed (*H*_o_) heterozygosity (Nei, 1987) and inbreeding (*F*_IS_) on neutral loci using Arlequin 3.5 (Excoffier & Lischer, 2010), GENEPOP 4.3 (Rousset, 2008), and GenAlEx 6.5 (Peakall & Smouse, 2012). We calculated pairwise and overall genetic differentiation (*F*_ST_) values (Weir & Cockerham, 1984) and levels of genetic variance between and among localities using the analysis of molecular variance (AMOVA) implemented in GENEPOP 4.3. The significance of these was tested using 1,000 and 999 random permutations in GENEPOP 4.3 and GenAlEx 6.5, respectively. All *p*‐values were adjusted for multiple comparisons test using the FDR method in the function “p.adjust” implemented in R v.2.15.2 with an experiment‐wide α = .01.

### 2.5 Identification of genetic clusters and localities under divergent selection

We identified genetically distinct groups of individuals (i.e., discrete populations) for putatively neutral SNPs and those under divergent selection using three methods, (1) Bayesian clustering, (2) Discriminant analysis of principal component (DAPC), and (3) Neighborhood‐joining phylogenetic tree. The Bayesian clustering approach implemented in STRUCTURE (Pritchard, Stephens, & Donnelly, 2000) identifies groups of individuals corresponding to the uppermost hierarchical level and has been shown to perform well with codominant markers such as SNPs. We used the correlated allele frequency and the admixture ancestry models without prior population information to assess values of *K* from 1 to 5. We performed 20 independent runs for 10,000 generations and 10,000 MCMC iterations for each value of *K*. The preferred value of *K* was determined using the change in the second order of likelihood, *ΔK* (Evanno, Regnaut, & Goudet, 2005) in Structure Harvester webserver (Earl, 2012). Discriminant analysis of principal component (DAPC) was carried out using the adegenet package v.1.4‐0 (Jombart, 2008) implemented in the R v.2.15.2. We ran DAPC for SNPs under neutral and divergent selection separately using the function “find.clusters.” We retained 80 and 20 principal components (PCs) for neutral and SNPs under divergent selection, respectively, as these explained the vast majority of genetic variation (Figure S3). Finally, we constructed neighborhood‐joining (NJ) phylogenetic trees on the putatively neutral SNPs and those under divergent selection using MEGA6 (Tamura et al., 2013). Simulations were performed based on Reynold's distance (Reynolds, Weir, & Cockerham, 1983) and bootstrapping of 10,000 replications over all loci.

Spatial patterns of divergent selection were inferred from the Neighborhood‐joining tree constructed using SNPs under divergent selection. Divergent selection creates a heterogeneous genomic differentiation by fixing adaptive traits, resulting in accentuated genetic divergence between locations affected by selection (Renaut et al., 2011). Genetic subdivision resulting from divergent selection will also show lower genetic variation between individuals within locations, than those locations under no selection (Renaut et al., 2011). Consequently, genetic subdivisions under strong divergent selection will yield on average an NJ tree with shorter and more‐uniform terminal branches and smaller overall length compared to those experiencing no or less selection pressures.

## 3 Results

### 3.1 Detection of loci under divergent selection

Among the three methods used, 138 (3.2%) loci were identified as candidates for divergent selection, 36 (0.84%) as under balancing selection, and the rest as putatively neutral. Fifty‐four of the loci under divergent selection were common to all three methods (Arlequin, Bayescan, and Lositan). Given that false positives are often associated with the outlier loci, we conservatively considered only these 54 loci for downstream analyses of adaptive genetic structure. Alignment of the trimmed sequences of the 54 SNPs (Table S2) to the list of nonredundant nucleotides in the UNI‐PROT/SWISS‐PROT and NCBI database provided a total of four hits with an *e*‐value less the 10^−6^. Of these, three carried a nonsynonymous SNP (Table 1). The SNP*3129* and SNP3*136* are situated in the gene NOS1, which encodes nitric oxide synthase 1, an enzyme that mediates biological processes, such as neurotransmission, antimicrobial, and antitumoral activities. In the central and peripheral nervous system, it is involved in neurotransmission (Hall et al., 1994). The SNP*3130* is situated in the FOXP2 gene, which encodes the Forkhead box protein P2 (Enard et al., 2002), the function of which is unknown in squamates. The SNP*3134* is situated in the MYH gene, which encodes the myosin heavy chain II isoform‐contractile proteins that modulate muscle contraction, cytokinesis, and phagocytosis (De La Cruz & Ostap, 2004).

**Table 1:** Characterization of high‐quality BLAST matches of four sequences of SNPs under divergent selection with nonredundant nucleotides in the NCBI database

SNP ID	Trimmed sequence	Gene	Species	E‐value	Hit length	Identity	Sequence ID	Gene ontology
SNP*3129*	CTGCAGGCTGGATTGGGGGTCTCTGCGGGCCACAAATGGCCCCCAGGCCAGGGTTTGCCCACCCATGCTC	NOS1	*Mabuya perrotetii*	6.00E‐14	489	90%	KJ574789.1	Encodes the enzyme nitric oxide synthase 1, which acts as a biologic mediator in several processes including neurotransmission, antimicrobial, and antitumoral activities
SNP*3130*	CTGCAGCCCCAAGGTAAGGGAACAAATGCTCCCATACCTTGAGGAGGTGTCTGTGACTACCTCCCAACCA	FOXP2	*Mabuya sp*.	2.00E‐07	845	81%	KJ574491.1	Unknown in squamates
SNP*3134*	CTGCAGCCCCAAGGTAAAGGAACAAATGTTCCCATACCATAAGGAGGCCTCTGGGACTGCTGCCCCACCA	MYH	*Mabuya sp*.	2.00E‐06	920	80%	DQ239423.1	Contains the ATPase activity providing energy that is the driving force for cytokinesis, phagocytosis, and muscle contraction
SNP*3136*	CTGCAGGATGCAGCACACGGCCCATTGGCACCGCTATGCCAGTGCTGGAAAGGAGTGTGCCCTAACAGTG	NOS1	*Eutropis novemcarinata*	2.00E‐08	715	88%	KJ574776.1	Encodes the enzyme nitric oxide synthase 1, which acts as a biologic mediator in several processes including neurotransmission, antimicrobial, and antitumoral activities

### 3.2 Genetic diversity and differentiation

Of the 4,100 putatively neutral loci, 357 (8.7%) deviated significantly (*p *<* *.01) from HWE, but only 115 (2.8%) remained significant after adjusting for FDR at α = .01. Expected heterozygosity (*H*_e_) varied across localities, ranging from 0.056 ± 0.002 at Sydney to 0.202 ± 0.03 at Armidale (mean ± *SE*). In general, *H*_e_ was not significantly different from the corresponding observed heterozygosity (*H*_o_), suggesting that the observed deviation from HWE was an artifact of sampling. Fixation index (*F*_IS_) was small and nonsignificant, except for samples from Sydney (*F*_IS_ = 0.263, *p *<* *.001) that were a combination of individuals from two isolated sites: Barrenjoey headland (33° 32′ S, 151° 20′ E) in the Kur‐ring‐gai Chase National Park and Box Head (33° 32′ S, 151° 19′ E) in Bouddi National Park (Table 2). Separate analysis of samples from these two sites showed no heterozygote deficit (Table S3), indicating that the high Fis observed when the samples were pooled is a Wahlund effect owing to genetic structure (Frankham et al., 2002). Genetic differentiation among all pairs of localities (i.e., pairwise *F*_ST_) was high and significantly different from zero in each case (*p *<* *.01), ranging from 0.126 (Bathurst vs. Crookwell) to 0.742 (Sydney vs. Crookwell) and averaging 0.542 over all populations and loci (Table 3). Analyses of molecular variance (AMOVA) indicated that genetic variation among populations accounted for 65% of population differentiation, while variation within individuals and between individuals accounted for 33% and 2%, respectively.

**Table 2:** Summary statistics (Sample size *N*, mean ± *SE* for observed [*H*_o_] and expected [*H*_e_] heterozygosity, inbreeding coefficient *F*_IS_) on neutral loci for the four sampling localities

Locality	*N*	*H*_o_	*H*_e_	*F*_IS_ (*p*‐value)
Armidale	27	0.192 ± 0.003	0.201 ± 0.003	0.0609 (.046)
Bathurst	27	0.129 ± 0.003	0.130 ± 0.003	0.0163 (.208)
Crookwell	22	0.126 ± 0.003	0.126 ± 0.003	0.0147 (.361)
Sydney	18	0.042 ± 0.001	0.056 ± 0.002	0.2631 (<.001)

**Table 3:** Pairwise population differentiation (*F*_ST_) for neutral SNPs (values below diagonal). Probability (*p*‐value) based on 9,999 permutations is shown above diagonal

Neutral Loci				
Locality	Armidale	Bathurst	Crookwell	Sydney
Armidale	0.000	0.001	0.001	0.001
Bathurst	0.644	0.000	0.001	0.001
Crookwell	0.655	0.126	0.000	0.001
Sydney	0.725	0.726	0.742	0.000

### 3.3 Delimitation of genetic clusters

**Figure 2:**
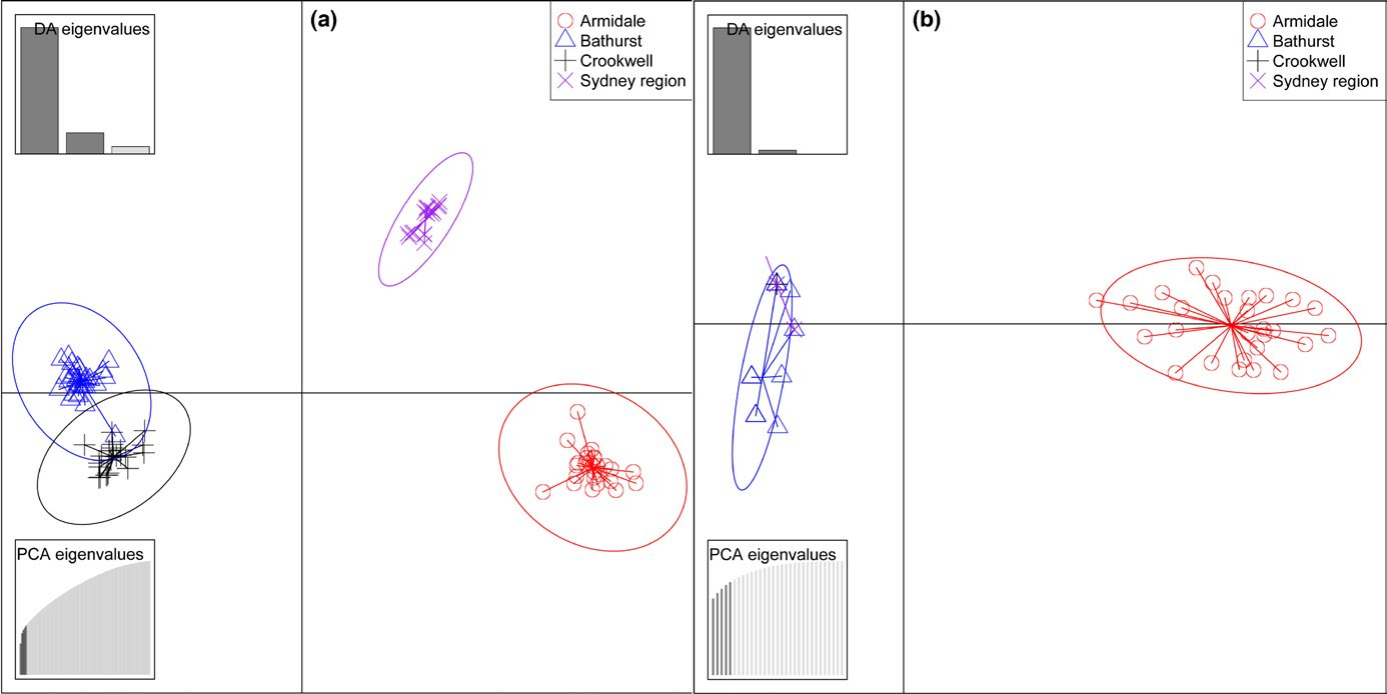
Discriminant Analyses of Principal Component (DAPC) on putatively neutral (a) and SNPs under divergent selection (b) showing three and two distinct population clusters, respectively

Bayesian clustering in STRUCTURE without prior locality information yielded a best‐fit value of *K *=* *3 on putatively neutral loci. Both DAPC and NJ trees identified samples from Armidale and Sydney as singletons (i.e., discrete populations that did not overlap with the other sampled populations) and those from Bathurst and Crookwell as overlapping clusters. For SNPs under divergent selection, two clusters were identified—Armidale as a singleton and Sydney, Bathurst, and Crookwell as the other (Figure 2), with strong fixation of alleles between these two clusters. Interestingly, all 4,274 SNPs (neutral plus outlier loci) identified patterns similar to the 3,851 putatively neutral SNPs, whereas all the SNPs identified as being under divergent selection by the individual methods showed similar spatial patterns as the 54 candidate SNPs for divergent selection that were common to all four outlier methods. This suggests that false positives had no significant influence on the results.

The NJ tree on SNPs under divergent selection showed uniform and short branch lengths for populations at Bathurst, Crookwell, and Sydney compared to those at Armidale. This suggested that these three populations experience stronger selection pressures than at Armidale.

## 4 Discussion

Our analysis of genetic structure in Cunningham's skink along the Great Dividing Range (GDR) in southeastern Australia revealed strong genetic partitioning and signatures of selection. Genetic structure at neutral SNPs and those under divergent selection yielded contrasting spatial patterns, showing that in this species, neutral genetic variation is not necessarily a good proxy for adaptive variation. At neutral loci, the genetic distances between each of the four localities analyzed were high, with a relatively high proportion of unique alleles at Armidale. SNPs putatively under divergent selection clustered into two groups (Armidale versus Sydney–Bathurst–Crookwell).

The level of genetic differentiation at neutral SNPs among individuals at Bathurst/Crookwell, Sydney, and Armidale locations was high (*F*_ST_ ≥ 0.644). This level of genetic partitioning is substantially above the *F*_ST_ level of 0.35, which is approximately the point at which the spread of advantageous alleles across a species’ range is prevented (Lowe & Allendorf, 2010). The levels of genetic divergence suggest that individuals at our study localities have long been isolated. Given the low movement rates characterized by this species (Stow & Sunnucks, 2004) and fragmentation of suitable rocky habitat across the study area, such high levels of genetic partitioning were anticipated.

While gene flow can aid the spread of advantageous alleles, high levels of gene flow can also stall local adaptations (Sexton et al., 2011; Slatkin, 1987). However, recent studies demonstrate that beneficial alleles can be maintained, favoured, and established, even under high gene flow (Schweizer et al., 2015; Sexton et al., 2014). Thus, neutral and adaptive genetic variation could show different spatial patterns even in highly dispersed species. For example, in the Atlantic herring (*Clupea harengus*) (André et al., 2011) and Atlantic cod (*Gadus morhua*) (Hemmer‐Hansen et al., 2013), neutral SNPs and those under divergent selection showed contrasting spatial patterns despite high gene flow.

In a heterogeneous and complex landscape, such as the GDR, species with low dispersal and gene flow among populations may show concordance between patterns of neutral and adaptive variation, if the limits of dispersal corresponds with clines in the environmental drivers of selection (Sexton et al., 2014). For example, in the relative philopatric anadromous Atlantic salmon (*Salmo salar*), similar spatial patterns at neutral and adaptive genetic variations have been reported (Moore et al., 2014). However, despite the limited gene flow and very high genetic structuring at neutral loci in Cunningham's skink, the neutral and adaptive genetic variations showed contrasting spatial patterns. While the neutral SNPs identified three clusters (Bathurst–Crookwell, Sydney, and Armidale), the putatively adaptive SNPs clustered into two groups (Armidale versus the other three localities). Although Sydney experiences different environmental conditions, and presumably different selection pressures from Bathurst and Crookwell, this was not detected in our dataset. This suggests that lizards at Armidale have been under different selection pressures of a greater magnitude.

We successfully aligned sequences of four of the SNPs identified to be under divergent selection with genes of known identity and function. This strongly supports the claim that these SNPs are located within a functional part of the genome. However, the *F*_ST_ outlier tests we used to identify loci under divergent selection are designed to detect “hard” selective sweeps that rapidly fix favorable alleles (Pritchard & Di Rienzo, 2010; Pritchard, Pickrell, & Coop, 2010). As a result, “soft” selection sweeps, which involve relatively small changes in allele frequencies at a large number of loci underlying the selected trait, may not have been identified (Brauer, Hammer, & Beheregaray, 2016; Pritchard & Di Rienzo, 2010; Pritchard et al., 2010). Ample evidence suggests that local adaptation to environmental change is largely via polygenic “soft” selection sweeps, that is, simultaneous selection acting on variants at many loci of small effects (Pritchard & Di Rienzo, 2010; Pritchard et al., 2010).

Our results have conservation implications under contemporary climate change. Shifts in climate could necessitate shifts in the distribution of the species and also change the locations where particular adaptive genes might be advantageous (Hannah, 2008). Historically, the level of connectivity (*F*_ST_ ≥ 0.644) is unlikely to allow for spread of favorable alleles (Lowe & Allendorf, 2010). The high *F*_ST_ values observed in our data also suggests that connectivity of populations may not be established given the rapid pace of climate change. If this is the case, the alternatives are *in situ* adaptation or localized extinctions.

Like all lizards, Cunningham's skinks are heliotherms, and to avoid overheating and death, the lizards must reduce their activity and retreat to cool refuges, reducing foraging time and constraining growth, maintenance, and reproduction (Sinervo et al., 2010). In addition to behavioral adaptation, lizards might be able to evolve a higher optimum body temperature, but this increases the risk of overheating as the optimum nears the critical maximum temperature (Sinervo et al., 2010). The constraint on thermal adaptation suggests that adaptation alone might not be enough to rescue some lizards from climate‐induced extinctions. For example, a positive correlation between the rate of increase in maximum air temperature of the coldest month and local extinctions has been reported among Mexican lizards (Sinervo et al., 2010).

Strong genetic structuring in Cunningham's skink complements similar findings reported in phylogeographic studies of other lizards, mammals, birds, and invertebrates inhabiting the GDR (Chapple et al., 2011; Pepper et al., 2014). For instance, the mean *F*_ST_ for geographic groups of two skink species was 0.96 for mtDNA and 0.89 for nuDNA in *Lampropholis robertsi*, and 0.70 for mtDNA and 0.8 for nuDNA in *L. coggeri* (Bell et al., 2010). Species distribution modeling under representative palaeoclimates suggested that these two species have existed along the GDR in multiple isolated populations throughout the climate cycles of the Pleistocene (Bell et al., 2010). Phylogenetic analysis of the common froglet, *Crinia signifera,* identified three geographically divergent lineages along the GDR that were separated during the late Miocene (~9 million years ago) (Symula, Keogh, & Cannatella, 2008). Three geographically separated clades of the lace monitor, *Varanus varius*, have also been identified, with divergences estimated to have occurred during the Pleistocene (Smissen et al., 2013). The high levels of population structuring for multiple taxa distributed along the GDR indicate a general pattern of long‐term isolation, predating recent anthropogenic habitat loss. This therefore suggests that habitat corridors may not be a universal solution for species needing to shift their distributions under climate change.

We provide the first genetic evidence for different selection pressures along the GDR for a vertebrate. The presence of divergent selection raises some concerns for alternative management strategies. Translocation is often proposed as a means to rescue species from potential extinction, particularly where the current range becomes unsuitable and there is little or no overlap between this and areas projected to be suitable in the future. Initiating gene flow raises the potential risk of outbreeding depression (Frankham et al., 2011), which can be the result of several factors, including genetic incompatibilities and disruption of co‐evolved gene complexes (Frankham et al., 2002). The presence of localized adaptation in Cunningham's skink highlights the potential for outbreeding depression to occur.

Unless climate change forces unprecedented levels of movement in Cunningham's skink, our data suggest that gene flow will be insufficient to spread advantageous alleles in the future. Knowledge of areas where divergent selection is associated with differences in climate can potentially be used to select areas for assisted migration purposes. However, studies on genetic structure based on neutral markers, such as those using microsatellites, may not be informative in this respect, because, as we have shown, patterns of variation at selected parts of the genome may not be concordant (Hemmer‐Hansen et al., 2013). In the case of the GDR, if translocation is to be adopted as a management tool, further knowledge on localized adaptation from other taxa will help evaluate whether patterns identified in this study are more general. Knowledge of the concordant patterns of selection across divergent taxa are likely to be crucial for the success of species recovery programs, if resources are not available for the genetic assessment of individual species.

## Conflict of Interest

None declared.
