# Supplementary material for: Cunningham's skinks show low genetic connectivity and signatures of divergent selection across its distribution
Source: Ecol Evol. 2016 Nov 29;7(1):48–57. doi: 10.1002/ece3.2627 (PMC5214970; doi:10.1002/ece3.2627)
Supplement: Supplementary file 1 [file ECE3-7-48-s001.docx]

## SUPPLEMENTARY INFORMATION

Text file 1.

## SNP discovery and screening

SNPs discovery and genotyping were performed at Diversity Arrays Technology Pty. Ltd. (Canberra, Australia) using standard DartSeq^TM^ protocol as follows:

First, 0.5 µL template DNA was incubated in a 1X solution of Multi-Core^TM^ restriction enzyme buffer (Promega) at 37°C for 2 hours to remove any nucleases present. The DNA was then run on a 0.8% agarose gel pre-stained with GelRed^TM^ at 120V for 20 minutes and checked visually for quality and quantity. Following the quality check, about 100 ng of each sample was transferred to a digestion and ligation reactor. Each sample was digested using a combination of *PstI* and *SphI* restriction enzymes and ligated with unique barcoded adapters (P1 adapter). Barcodes were designed to alter the enzyme recognition sites so that heat killing of enzyme was not required.

Digested and ligated samples were cleaned using a spin-column Qiagen PCR clean up kit and then amplified using PCR with barcode- and adapter-specific primers. Cleaned PCR product was then ran on 0.8% agarose gel to check that digest fell within the standard range. Equimolar amounts of amplified samples from each individual was pooled into a single tube, diluted and denatured using NaOH and then hybridized to the flow cell. The library was then sequenced with Illumina HiSeq2500 (Illumina) for 77 cycles, resulting in fragments of 77 bp long. To assess the reproducibility of SNPs calls, ≥ 15% random replicates were carried through the protocol pipeline. Once sequencing was completed, raw sequenced data were converted to .fastq files using the Illumina HiSeq2500 software. Sequences from each sample were collected, separated by individuals, stripped of barcodes, cleaned and filtered to include only those with a Phred score ≥ 25. SNPs were identified and called following standard protocols in DArTSoft14^TM^ (Diversity Arrays Technology). Initial alignment to existing sequences in the Dart database from similar organisms was performed to identify any contamination. Alignments to viral and bacterial sequences were also performed to further identify contamination. All monomorphic sequence clusters were removed and SNPs were called only if they were present in both homozygous and heterozygous forms.

**Table S1:** Matrix of average pairwise geographic distance (km) between sampled localities.

| **Locality** | **Armidale** | **Bathurst** | **Crookwell** | **Sydney** |
| --- | --- | --- | --- | --- |
| **Armidale** | 0 |  |  |  |
| **Bathurst** | 380 | 0 |  |  |
| **Crookwell** | 460 | 96 | 0 |  |
| **Sydney** | 310 | 192 | 212 | 0 |

**Table S2:** Sequence of the 54 SNPs identified by all three genome scan methods to be candidates for divergent selection

| **SNP ID** | **SEQUENCE** |
| --- | --- |
| SNP3117 | CTGCAGTTTTTCCCTTTACTGTTTTTCAAAGGAACAATGTGCATG |
| SNP3118 | CTGCAGGCTCCACATGATTGGGGTCCCCTGTTCTATAGCATG |
| SNP3119 | CTGCAGTCCAAAGGCCTTGTGAGCGTGCAGAAGATCTGCATG |
| SNP3120 | CTGCAGTGGCTCTGAAGGAGCGGGGAAAGGGCCGCTTGCATG |
| SNP3121 | CTGCAGTCCTCACCCACTTTCCAGAGAGTAAGCCCCTTGGGCGATAATGGGGGCTTGCTTCTGATGGGGC |
| SNP3122 | CTGCAGCGACAACATGCAGAACATGGCTGCCAACACAAAAGACTGGTGAGGGTGCGTGCATG |
| SNP3123 | CTGCAGGACTGTCTTGCAATACCACCCTGAATCCAAGCCAAGTAATAGAGTAGGAACCCAATCCTATGTT |
| SNP3124 | CTGCAGAGGCTTGTTCTCAACAGTGGTATGCAGTGTGGCACAGGTGCTGCTGGTGAGAGAGTGGAATTCT |
| SNP3125 | CTGCAGGTCGCTATGTGTGCGCGCATG |
| SNP3126 | CTGCAGCCTCTACTTGCACAGCAAGGAAGCTGATGGAGGTGAAGAATAAGCAAGGGCTGCATGAGATCGG |
| SNP3127 | CTGCAGCTAGAGGGGACAACTCAGGTAATGTTAACATGAAGCAGGAAGCACATTTGCTGCCTGCATGAGA |
| SNP3128 | CTGCAGCTCCCCAGCTCTGGATATTTTCTTCCTAATCTTGAGTTATCAGTGGGGCAAATCAACTTGCAAA |
| SNP3129* | CTGCAGGCTGGATTGGGGGTCTCTGCGGGCCACAAATGGCCCCCAGGCCAGGGTTTGCCCACCCATGCTC |
| SNP3130* | CTGCAGCCCCAAGGTAAGGGAACAAATGCTCCCATACCTTGAGGAGGTGTCTGTGACTACCTCCCAACCA |
| SNP3131 | CTGCAGTGCTACCATTTGTTTCTTGATTCCTTTAGCACCAGCATTACACAGAAATAGCTTTACCCGTCTT |
| SNP3132 | CTGCAGGGTACACTATGACTCGTGCCTTCAGTCGCAGGGTCGCTGACTTGTGACTTGACTTGGGAGACAG |
| SNP3133 | CTGCAGGGAAGCCATCCTTCCCTGTGGGCCAAACTGCACTTTGGCCTGCCACTTCCTTGTTCCATTGCCC |
| SNP3134* | CTGCAGCCCCAAGGTAAAGGAACAAATGTTCCCATACCATAAGGAGGCCTCTGGGACTGCTGCCCCACCA |
| SNP3135 | CTGCAGTAGTGAGTCTTGTGATCTGCTGCCGTAAGACCAGTTATGGTGCACAAAAGGTGCACTTCCATTG |
| SNP3136* | CTGCAGGATGCAGCACACGGCCCATTGGCACCGCTATGCCAGTGCTGGAAAGGAGTGTGCCCTAACAGTG |
| SNP3137 | CTGCAGCTCTCAGGAACCATTTCCTGTACCTAAGCCACAGTAGGAAAAGCTGGGAAATCTCATTTGGGAA |
| SNP3138 | CTGCAGCTGCAATGGGTCAAATCAGACCTGTGCCGACTCTATAGCTGGCACAAGTCTGTGTTGATCTGTG |
| SNP3139 | CTGCAGGTGTGCCTTGGGAGTTTGGGGAATGGTCATTTATTAATAGGGCCATTGGGGAATGTGAGCTAGC |
| SNP3140 | CTGCAGTCTATTCTTGTAATGAAAATAGAAGTTTTCACTTTTCTTCTCTGACACGTGAGGAAAAAACAGA |
| SNP3141 | CTGCAGCCACCTCTGCACTTTGTTTAACCCTGTTGTCTATTTCTGGGCATGGGGTCTAGTATAGTTTCAC |
| SNP3142 | CTGCAGTGATATGAGAAATGGCACCTGGTAGAATGTTCACTTCCATGTGCATCTCAAGGTACAGGAACTC |
| SNP3143 | CTGCAGCAACTCTCCCCAGAGTAAGGGGAAGTGATTCCCCTTGCCCTGGGCTGAACCGCAACTAGCCCCA |
| SNP3144 | CTGCAGGTTGGGGGCTGTATGTTAGAATGTTCCACCAGGTAAAGAAGTCTCCCTGCATGTAGGCAAGTAG |
| SNP3145 | CTGCAGATTTTCACCCTGGTCATTCAGTACAACTTGTGCCCACCTAGCTACTAACAGAGCAGGCATGAGA |
| SNP3146 | CTGCAGTCCCACGCACTGGGAAGCTCAGGATTGGGCTGCCCATCAGCTAGGGAGATACATGTGAAGAAAC |
| SNP3147 | CTGCAGTGTTAGGAACTTGGCAGCATG |
| SNP3148 | CTGCAGGAAGAGGGGTTATCTGTTCAAATGCTTCTGCCTACCCACTGATCCAGCTGGCCACATGCATGAG |
| SNP3149 | CTGCAGGATTAGTATGTGGATTTTTATAATGGCACACGCACATTATATATGCATG |
| SNP3150 | CTGCAGCAGGAATTATTAAGGGGAAAGACCATGGCCCAGTGACGTATGGCTCTTGTGCTTTGCATGAGAT |
| SNP3151 | CTGCAGACACTGGACTGTATGTGCATAGGTTGTATTTAAGCTTTATGTTCCTGGGTGGACTGAGAGACGT |
| SNP3152 | CTGCAGTTGTATCAAGCCGTTCATGGTTTTTGTAGTCTTTTGGCTGTCCTTGGTAATATGGTATCAGGAT |
| SNP3153 | CTGCAGGCCCCTAGTCTTAGCAGTAAGTCTTTGATGTTGCTGATGTTTTGTGCCAGTGGCTCAGAAATGG |
| SNP3154 | CTGCAGTGAGGTGGCCTGAGGCCCGGGACATAGAGCCTGCTCTCGTTCCTTCCACTGTGTCATAGCAATT |
| SNP3155 | CTGCAGTGGAGGGACTGTCTGGAGGCCTCCTTGAGATAAGGGAACTTTCATTCCCTTACCCTGGGGAAAG |
| SNP3156 | CTGCAGAAATTATAGTTGCCTCTTTACTCAGTTAATTAGTAGATAACTCAAGCAAGCCATCAGGAAATAC |
| SNP3157 | CTGCAGCAAAATGGACCCAGGTGATTCCGTAAAATTGGCTGTACAGCTCTGAGTGCTCTCTTAAGAGATG |
| SNP3158 | CTGCAGCACAAGGTAGATCGTGGCATGGGCAAGAGTGGGCACAAGAGGATGTGCTCTAATTGCTTCCCCA |
| SNP3159 | CTGCAGTCTATAGACTAGTTAATATATCCCCGTGGTGGCTTCAGTCTGGGCCATAAAGTGCGAAGTGGGG |
| SNP3160 | CTGCAGGGAGAAGTTTCACAACTAGCCAGCCAGCATGTCACTCAGTCAGGCACGAAGGCTGCCGCTGGGT |
| SNP3161 | CTGCAGCAGTCAGATACATGCACACATATGACAGACTGGCACTGCGATGCACCTTACACCACATGCACAC |
| SNP3162 | CTGCAGCCTTGCACACAATCACTAAAGACTGTGCGTGTGCAGGATGACTGTTATAGCCATTCCAGTGCAA |
| SNP3163 | CTGCAGGTCAGCCAGACAGCATGGGACATTGGAAATGGCCGGTAATTGGCTCTGCTCTGTGAGTAGAGCT |
| SNP3164 | CTGCAGCCTCACATGGCAGTTTTGCTGTTGGCCAGCAGGCAGCCCACACTGCCCCACAGAGTACCTCGTA |
| SNP3165 | CTGCAGAACAAATCCAGTGTTTTGTACTGGTCAGTGACTGTAATAAAAGATTCTTCAATTCAATCCAGTG |
| SNP3166 | CTGCAGTGGGTGGGGAGGCAGAGCTTCTTCACTGGAGTCCTTCAAAAACTGCTGCTGCTATGTGAGGCAC |
| SNP3167 | CTGCAGCATTAGAATATTTAGTTCAACCGTTTTCAGACTGAGAAATATGTACAGATCTGGTGGGAGAAGC |
| SNP3168 | CTGCAGAGCTTCTGGTGTGTGTTCTAGGATACAAATTTGGTTCATGTGGAGCACAGACCTGTTGACCCTT |
| SNP3169 | CTGCAGTTCAGTTTTAGTTAAAACAGTGAAAAGGTGACTTTTTGAGACATGTCTTTTGAGGGTGGCTGCA |
| SNP3170 | CTGCAGACCACTGGGACATATTTGGATTGGCGCATAAGTTATTTGATTCTTAAGCAAGAAATTAGCAATA |

**Table S3:** Summary of F statistics (Sample size N, mean ± standard error observed heterozygosity Ho, expected heterozygosity He, inbreeding coefficient Fis) on neutral loci for Sydney Barrenjoey Headlands (BJ) and Box Head (BH).

| **Locality** | **N** | **Ho** | **He** | **Fis (*P*-value)** |
| --- | --- | --- | --- | --- |
| **Sydney BJ** | 11 | 0.039±0.003 | 0.036±0.003 | - 0.0269 (0.706) |
| **Sydney BH** | 7 | 0.048±0.003 | 0.041±0.003 | -0.0119 (0.947) |
